# Cytokine Regulation in Human CD4 T Cells by the Aryl Hydrocarbon Receptor and Gq-Coupled Receptors

**DOI:** 10.1038/s41598-018-29262-4

**Published:** 2018-07-19

**Authors:** Jeremy P. McAleer, Jun Fan, Bryanna Roar, Donald A. Primerano, James Denvir

**Affiliations:** 1 Department of Pharmaceutical Science and Research, Marshall University School of Pharmacy, Huntington, WV 25755 USA; 2 Department of Biomedical Sciences, Joan C. Edwards School of Medicine, Marshall University, Huntington, WV 25755 USA

## Abstract

Th17 cells contribute to host defense on mucosal surfaces but also provoke autoimmune diseases when directed against self-antigens. Identifying therapeutic targets that regulate Th17 cell differentiation and/or cytokine production has considerable value. Here, we study the aryl hydrocarbon receptor (AhR)-dependent transcriptome in human CD4 T cells treated with Th17-inducing cytokines. We show that the AhR reciprocally regulates IL-17 and IL-22 production in human CD4 T cells. Global gene expression analysis revealed that AhR ligation decreased *IL21* expression, correlating with delayed upregulation of *RORC* during culture with Th17-inducing cytokines. Several of the AhR-dependent genes have known roles in cellular assembly, organization, development, growth and proliferation. We further show that expression of GPR15, GPR55 and GPR68 positively correlates with IL-22 production in the presence of the AhR agonist FICZ. Activation of GPR68 with the lorazepam derivative ogerin resulted in suppression of IL-22 and IL-10 secretion by T cells, with no effect on IL-17. Under neutral Th0 conditions, ogerin and the Gq/11 receptor inhibitor YM254890 blunted IL-22 induction by FICZ. These data reveal the AhR-dependent transcriptome in human CD4 T cells and suggest the mechanism through which the AhR regulates T cell function may be partially dependent on Gq-coupled receptors including GPR68.

## Introduction

CD4 T helper cells direct immune responses by differentiating into specialized subsets named Th1, Th2, Th17 and regulatory T cells (Tregs)^1^. The balance of subsets generated in response to the cytokine milieu profoundly influences inflammatory disease outcomes. Although CD4 T cells are classified by their effector cytokines (Th1/IFN-γ, Th2/IL-4, Th17/IL-17, Treg/IL-10), it is now understood that they are plastic and retain the potential to differentiate into other subsets^2^. The multi-functional potential of CD4 T cells along with their antigen specificity makes them attractive therapeutic targets.

Th17 cells contribute to host defense against bacteria and fungi on mucosal surfaces but may induce chronic inflammatory diseases when directed against innocuous antigens^3^. The differentiation of naïve CD4 T cells into effector Th17 cells in lymph nodes is facilitated by antigen, IL-6, TGF-β, IL-1β and IL-23, resulting in the production of IL-17. Some Th17 cells also produce IL-22, IL-10 or IFN-γ which can have pro- or anti-inflammatory properties^4,5^. The receptors for IL-17 and IL-22 are primarily localized to mucosal surfaces including the gastrointestinal (GI) tract and lungs^6,7^. While IL-17 stimulates G-CSF secretion from epithelial cells leading to neutrophil recruitment, IL-22 induces antimicrobial peptide secretion and epithelial repair following injury^8^. Several models have demonstrated a role for IL-17 in chronic inflammation^3^. On the other hand, IL-22 and IL-10 protect against colitis^9,10^. Thus, there is considerable interest in understanding how pro- and anti-inflammatory cytokines are regulated in human Th17 cells.

The aryl hydrocarbon receptor (AhR) is activated by many endogenous ligands and natural products that have disparate effects on inflammation and T cells^11^. During Th17 cell differentiation, the AhR is upregulated and can increase production of the effector cytokines IL-17 and IL-22^12^. Notably, the AhR ligands FICZ or 2,3,7,8-tetrachlorodibenzo-p-dioxin (TCDD) can induce Th17 or Treg differentiation, respectively, resulting in increased or decreased susceptibility to experimental autoimmune encephalomyelitis^13^. The mechanism underlying pro- versus anti-inflammatory effects of AhR activation in T cells remains unclear.

Proton-sensing G-protein-coupled receptors (GPR4, 65, 68, 132) are heterotrimeric complexes that sense extracellular changes in pH^14^. Ischemia and chronic inflammation promote extracellular acidification through the stimulation of anaerobic glycolysis. The activation of proton-sensing GPRs can lead to the expression of inflammatory mediators including COX-2, prostaglandins and cytokines^14^. GPR68 is expressed in several cell types including the immune system and transmits signals through G_q/11_ proteins under acidic conditions, leading to the activation of phospholipase C (PLC), inositol triphosphate and intracellular Ca^2+^ mobilization. GPR68 is fully active at pH 6.8^15^. Notably, G_q/11_ signaling regulates murine Th17 responses *in vivo*^16^, suggesting a potential role for the GPR68/G_q/11_ pathway in T cell differentiation.

We examined the AhR on human CD4 T cells cultured with or without Th17-inducing cytokines. In contrast to mouse studies^12,13^, FICZ suppressed IL-17 production from human CD4 T cells. To study the mechanism of AhR regulation of cytokine production, we performed RNA-seq on T cells derived from peripheral blood of six healthy human volunteers treated with an agonist or antagonist. The AhR regulated several genes involved in cellular assembly, organization, development, growth and proliferation. The expression of G-protein-coupled receptor 68 (GPR68) positively correlated with IL-22 and was studied further. Activation of GPR68 with a positive allosteric modulator suppressed IL-22 and IL-10 production from human CD4 T cells cultured with Th17-inducing cytokines. Under neutral Th0 conditions, ogerin and the G_q/11_ inhibitor YM254890 partially suppressed IL-22 induction by FICZ. These data suggest that pro- versus anti-inflammatory effects of AhR may be partially regulated by G_q/11_ receptors including GPR68. We speculate this pathway may regulate inflammation in peripheral tissues with extracellular acidification.

## Results and Discussion

### Time course of human Th17 cell gene expression

**Figure 1:**
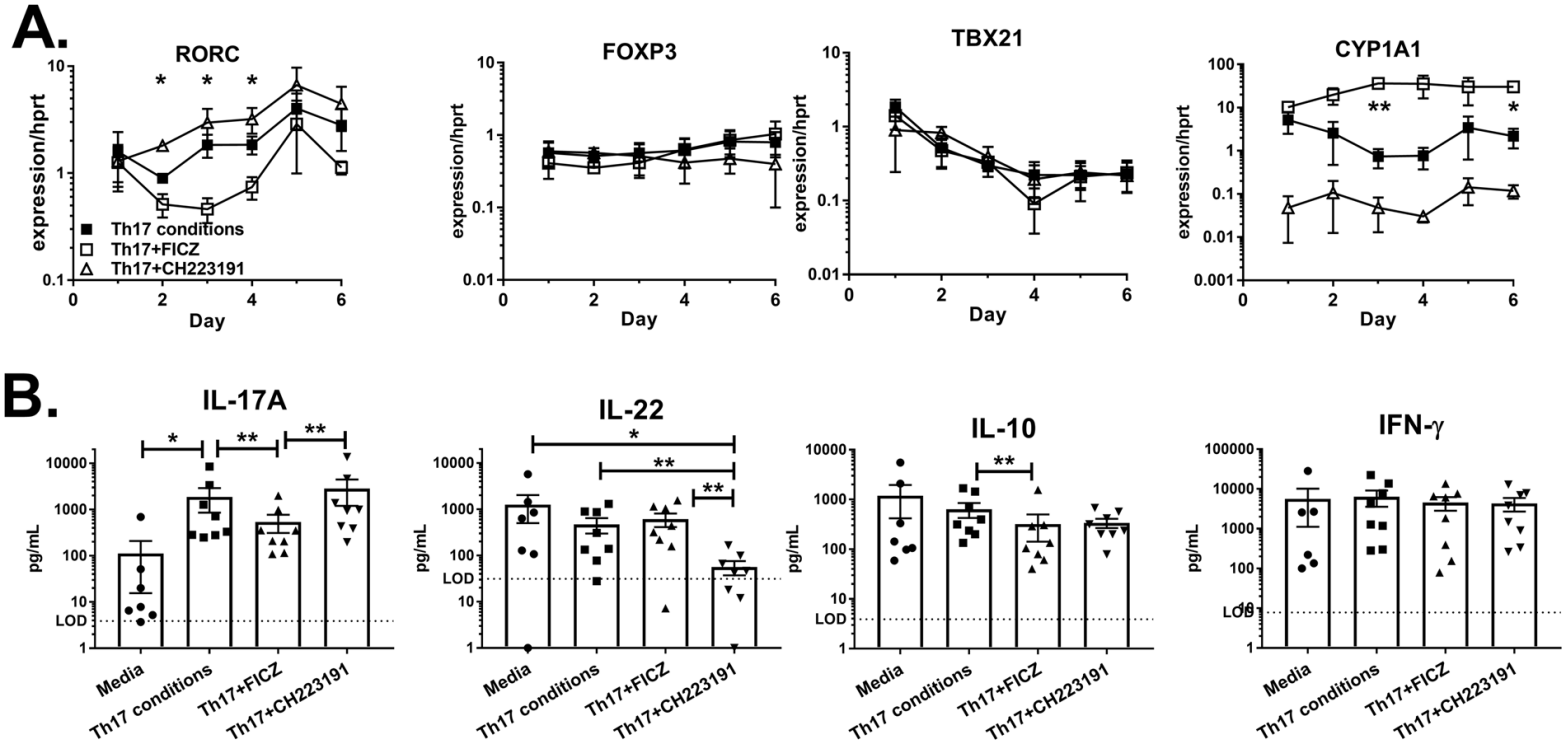
Effect of AhR modulators on human CD4 T cell differentiation. Naïve human CD4 T cells were cultured with Th17-inducing cytokines (IL-1β, IL-6, IL-23, TGF-β) in the presence or absence of FICZ or CH223191 for 1–8 days. (**A**) Gene expression in cultures at daily intervals, normalized to the housekeeping gene *HPRT*. Data are combined from 3 experiments (n = 3) and shown as mean+/− SEM. (**B**) Cytokine levels in culture supernatants on days 6–8 measured by ELISA. Data are combined from 8 experiments (n = 7–8). LOD = limit of detection.

To determine the kinetics of gene expression associated with human Th17 cell differentiation in the presence of AhR modulators, naïve CD4 T cells from peripheral blood of healthy volunteers were cultured with Th17-inducing cytokines (IL-6, TGF-β, IL-1β, IL-23) for 1–6 days. Serum-free RPMI was used as the cell culture medium in order to minimize AhR activation in control groups^4^. Although optimal Th17-differentiation conditions include neutralizing antibodies against IFN-γ, IL-2 and IL-4^4,17^, we chose not to use neutralizing antibodies in order to test if AhR activation of human CD4 T cells promotes differentiation into non-Th17 subsets. These conditions resulted in heterogeneous T helper cell populations in our cultures. Experimental cultures were treated with the AhR agonist FICZ (200 nM) or antagonist CH223191 (4uM). Th17-inducing cytokines resulted in a sustained increase in expression of the AhR target gene *CYP1A1* compared to freshly isolated naïve CD4 T cells (Fig. 1A). The addition of FICZ to Th17 cultures further increased CYP1A1 by an order of magnitude, while CH223191 potently suppressed *CYP1A1*. Thus, treating CD4 T cells with FICZ or CH223191 resulted in AhR activation or inhibition, respectively. Expression of the master Th17 cell transcription factor *RORC* decreased by 50 percent between days 1 and 2 of culture, followed by a 2-fold increase between days 2 and 3 (Fig. 1A). *RORC* expression peaked on day 5 at levels 4.5-fold higher than observed on day 2. FICZ delayed the upregulation of *RORC* on days 3 and 4, consistent with a suppressive effect on Th17 cell differentiation. In the presence of Th17-inducing cytokines, treatment with CH223191 prevented the downregulation of *RORC*, resulting in a 40 percent increase between days 1 and 2 (Fig. 1A). This effect was dependent on Th17-inducing cytokines, as *RORC* was downregulated from days 1–4 in Th0 cultures with CH223191 (Supplementary Fig. S1). These data suggest that the activated AhR can delay *RORC* upregulation during human Th17 cell differentiation. This effect was not associated with conversion to a regulatory T cell (Treg) or Th1 cell phenotype, as *FOXP3* and *TBX21* expression were not significantly affected by the AhR modulators in the presence of Th17-inducing cytokines (Fig. 1A).

### Reciprocal regulation of IL-17 and IL-22 secretion by human CD4 T cells through the aryl hydrocarbon receptor

Next, cytokines were measured in culture supernatants on days 6–8. Control cultures incubated without additional differentiation cytokines produced very little IL-17 (Fig. 1B, Media). Treatment with Th17-inducing cytokines significantly increased IL-17 secretion (1881pg/mL +/− 1020). Addition of FICZ to Th17 cultures reduced IL-17 by 71 percent on average (539 pg/mL +/− 229), demonstrating that the AhR inhibits Th17 differentiation. This inhibitory effect of FICZ was also observed for IL-10 production (Fig. 1B). In contrast, the inhibitor CH223191 resulted in a trend towards increased IL-17 secretion. Although FICZ has been shown to increase IL-22 production from human CD4 T cells^18^, the addition of FICZ to cultures containing Th17 inducing cytokines did not significantly increase IL-22 (Fig. 1B). Pairwise analysis revealed that FICZ increased IL-22 in six out of eight donors. Further, FICZ significantly increased IL-22 in the absence of exogenous cytokines (Supplementary Fig. S2). It has been reported that TGF-β suppresses IL-22 and AhR function^19–23^. To determine if TGF-β suppresses IL-22 induction by FICZ, T cells were cultured with IL-6, IL-1β, IL-23 and the TGF-β receptor I inhibitor Galunisertib. These “Th22” conditions significantly increased IL-22 production compared to culture with Th17-inducing cytokines (Supplementary Fig. S2). Notably, FICZ had no effect on IL-22 when T cells were cultured with exogenous cytokines, suggesting that AhR stimulation did not synergize with inflammatory cytokines.

**Figure 2:**
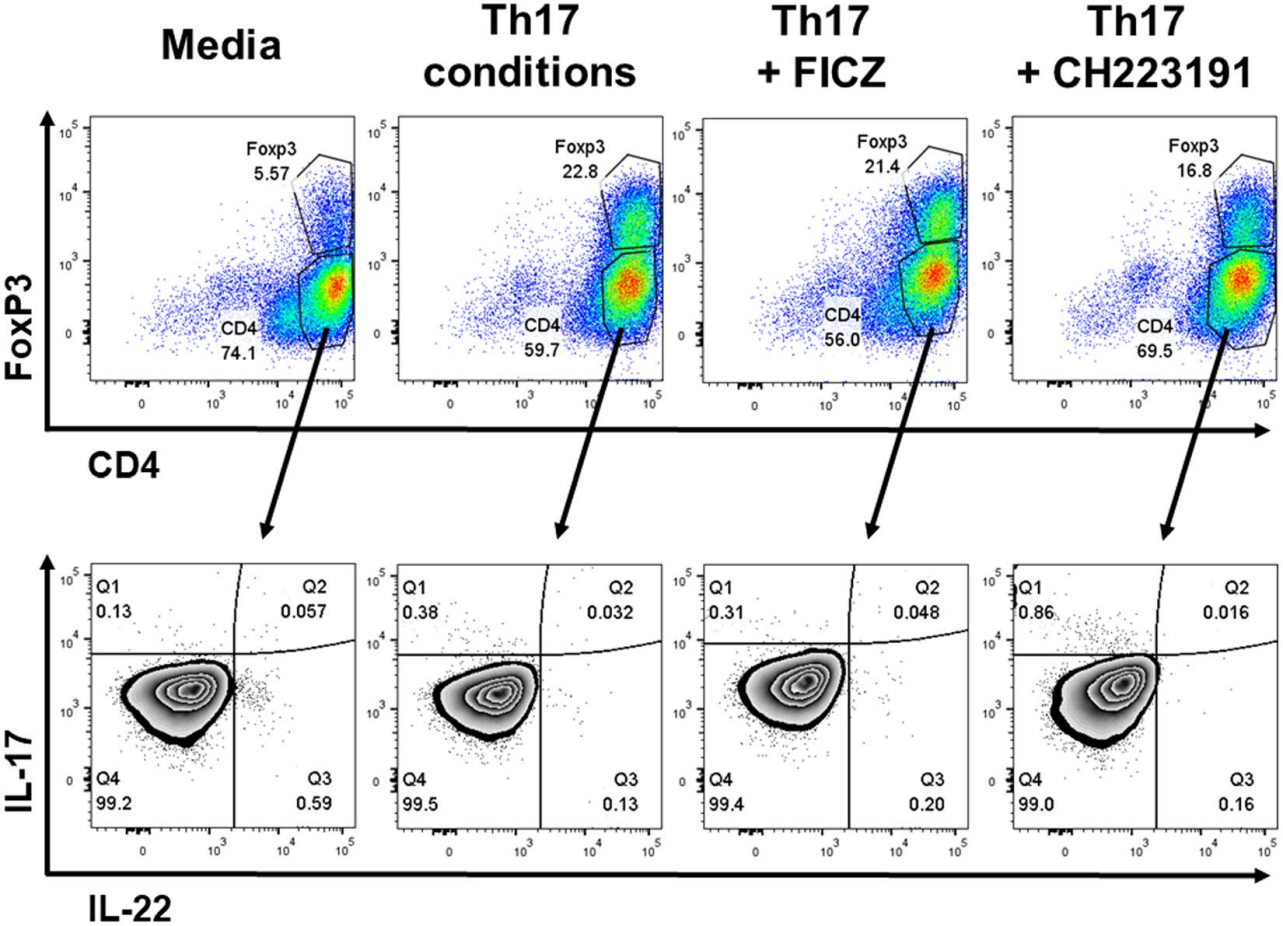
CD4 T cell phenotype in serum-free RPMI. Human peripheral blood naïve CD4 T cells were purified by negative selection and cultured for five days with plate-bound anti-CD3 and soluble anti-CD28. Additional treatment groups included Th17-inducing cytokines (IL-1β, IL-6, IL-23, TGF-β), AhR agonist (FICZ, 200 nM) or AhR antagonist (CH223191, 4 uM), as indicated. On day 5, T cells were restimulated with PMA and ionomycin in the presence of brefeldin A for 4 hours, stained with fluorescent-labelled antibodies and analyzed by flow cytometry. Shown are representative dot plots of CD4 versus FoxP3 on live cells (top) or IL-22 versus IL-17 on CD4^+^ FoxP3^−^ cells (bottom).

Nevertheless, the AhR was required for IL-22 production, as CH223191 potently decreased IL-22 levels by 88 percent. Flow cytometric analysis revealed that IL-17 and IL-22 were produced by distinct cell populations, as there were very few IL-17^+^ IL-22^+^ double-producing cells (Fig. 2). The frequency of cells producing either IL-17 or IL-22 was less than one percent due to culturing under suboptimal Th17 differentiation conditions in serum-free RPMI; thus, it is likely that the majority of IL-17 and IL-22 detected by ELISA (Fig. 1B) was derived from ~10^3^ cells. Overall, early changes in gene expression caused by AhR modulators (Fig. 1A, days 1–3) correlated with later effects on cytokine production (Fig. 1B, days 6–8), suggesting the AhR regulates early stages of Th17 differentiation.

### CD4 T cell transcriptome during culture with Th17-inducing cytokines and AhR modulators

**Figure 3:**
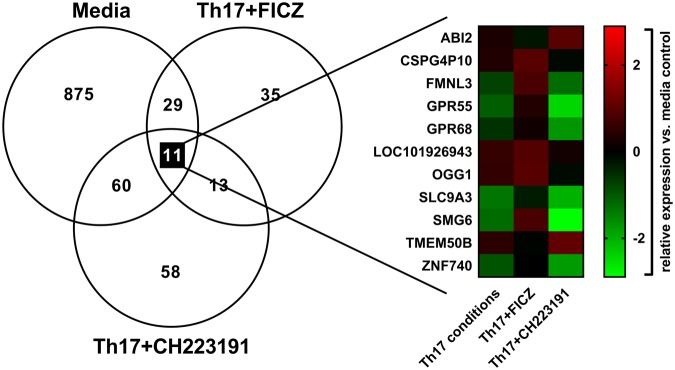
Differentially expressed genes in Th17 cell cultures on day 3. Naïve human CD4 T cells were cultured under four conditions for three days (media, Th17-inducing cytokines (Th17 conditions), Th17 + FICZ, Th17 + CH223191), followed by RNA-seq. Left: Venn diagram showing the number of differentially expressed genes for each condition compared to the Th17 group. Overlapping areas of circles represent genes regulated by multiple treatments. Right: Heat map showing relative expression of the genes regulated by all three treatments compared to the media control.

To gain insight into the mechanism of how AhR regulates human Th17 cell differentiation, we performed RNA-seq on day 3 cultures in the presence or absence of AhR modulators. This time point was chosen because induction of the Th17 cell marker *RORC* was first observed on day 3 and delayed by FICZ treatment (Fig. 1A), suggesting the AhR regulates early Th17 cell gene expression. Supernatant cytokines were approximately 10-fold lower on day 3 (Supplementary Fig. S3) compared to days 6–8. At this time point, Th17-inducing cytokines significantly increased IL-17 and IFN-γ production; however, the addition of FICZ or CH223191 had no effect on cytokines measured in day 3 culture supernatants (Supplementary Fig. S3). Illumina TruSeq mRNA libraries were prepared from total RNA and sequenced using the Illumina HiSeq1500 platform. Data from six individual donors were pooled and differentially expressed genes between each treatment and the control were identified (Fig. 3). In total, 975 genes were significantly up- or down-regulated in cultures with Th17-inducing cytokines compared to media controls cultured with anti-CD3 and anti-CD28 only. Ingenuity pathway analysis revealed that most of these genes were involved in cell death or survival, gene expression, cell cycle or protein synthesis (Table 1). The addition of FICZ to cultures with Th17-inducing cytokines altered the expression of 88 genes, while Th17 + CH223191 treatment significantly affected 142 genes compared to the Th17 group. Notably, the AhR agonist FICZ regulated genes involved in small molecule biochemistry and lipid metabolism, while the AhR inhibitor CH223191 primarily affected the expression of genes involved in cellular assembly, organization, development, growth, proliferation and cell cycle. Several genes were co-regulated by 2 or more treatments (Fig. 3, Venn diagram overlapping regions of circles).

**Table 1:** Most common cellular functions for genes differentially expressed by each condition relative to Th17 treatment.

Media		p-values	Th17 + FICZ		p-values	Th17 + CH223191		p-values
Cell function	Genes		Cell function	Genes		Cell function	Genes	
Cell death and survival	319	4.6 × 10^−42^ – 1.9 × 10^−5^	Small molecule biochemistry	10	7.8 × 10^−6^ – 5.0 × 10^−2^	Cellular assembly and organization	18	5.9 × 10^−5^ – 9.0 × 10^−3^
Gene expression	223	1.3 × 10^−16^ – 9.6 × 10^−6^	Lipid metabolism	5	7.8 × 10^−6^ – 3.3 × 10^−2^	Cellular development	15	2.0 × 10^−5^ – 8.9 × 10^−3^
Cell cycle	168	9.7 × 10^−14^ – 2.0 × 10^−5^	Nucleic acid metabolism	3	2.2 × 10^−4^ – 2.8 × 10^−2^	Cell cycle	14	5.9 × 10^−5^ – 9.2 × 10^−3^
Protein synthesis	108	3.0 × 10^−22^ – 9.6 × 10^−6^	Vitamin and Mineral Metabolism	3	1.6 × 10^−4^ – 3.6 × 10^−2^	Cellular growth and proliferation	14	3.0 × 10^−5^ – 9.2 × 10^−3^
RNA post-transcriptional modification	60	1.2 × 10^−22^ – 2.2 × 10^−6^	Drug metabolism	2	2.4 × 10^−5^ – 2.8 × 10^−2^	Cell morphology	10	2.0 × 10^−5^ – 8.9 × 10^−3^

Numbers of differentially expressed genes in functional categories, identified by Ingenuity Pathway Analysis. This platform reports p-values for the overlap of specific subcategories of each functional category under the null hypothesis that the set of differentially expressed genes is independent of the subcategory. The range of p-values over these subcategories is indicated.

**Figure 4:**
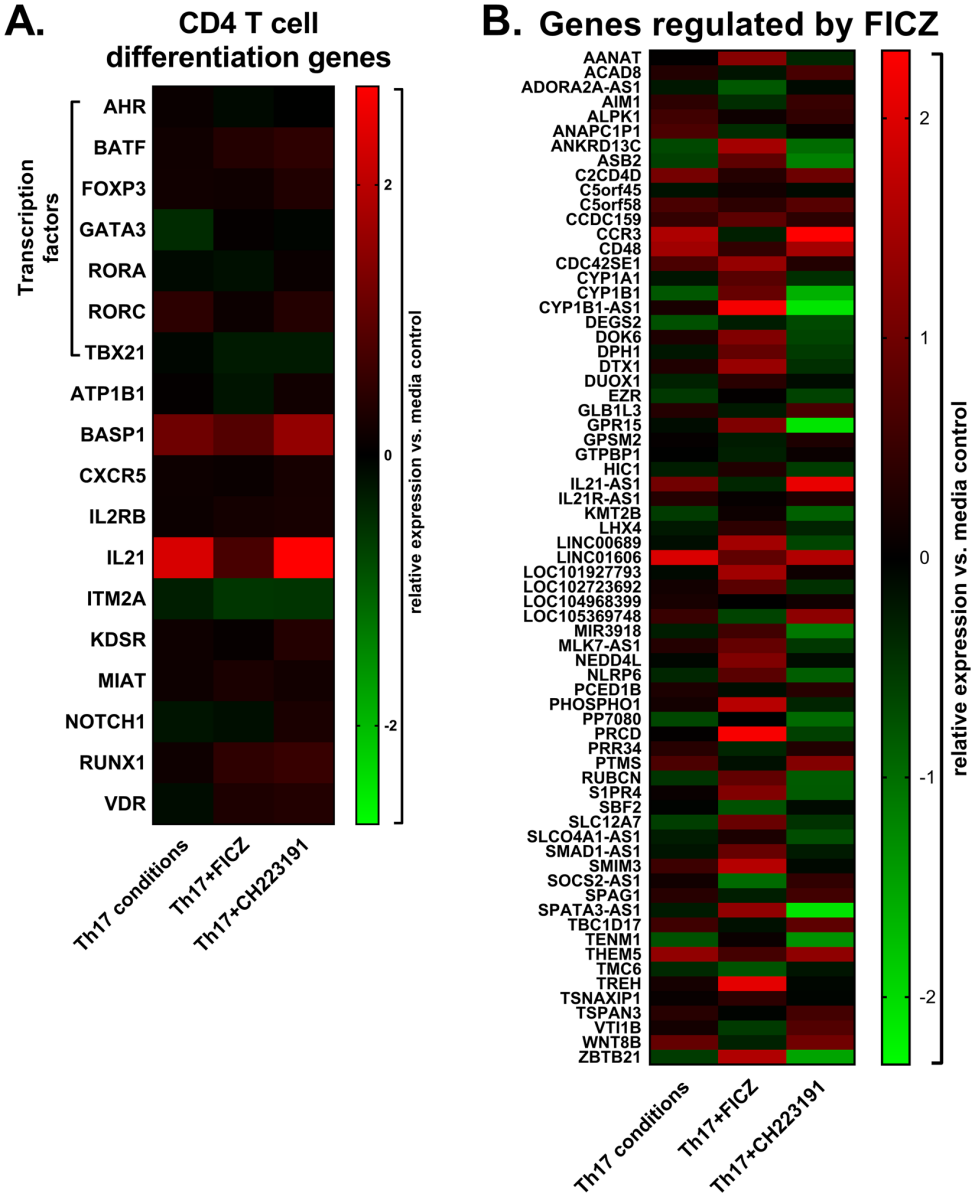
Early Th17 cell gene expression in the presence of AhR modulators. (**A**) Heat map of selected genes associated with CD4 T cell differentiation for each treatment relative to the media control. (**B**) Heat map of genes significantly regulated by FICZ compared to cultures containing Th17-inducing cytokines (Th17) without FICZ.

Many genes with known roles in CD4 T cell differentiation were not significantly affected by treatments on day 3 (Fig. 4A); however, Th17-inducing cytokines resulted in a significant increase in *IL21*, encoding for an autocrine cytokine required for Th17 differentiation^24,25^. FICZ treatment prevented the upregulation of *IL21* expression (Fig. 4A). This was associated with a modest decrease in *RORC* and increase in *GATA3* expression compared to cultures treated with Th17-inducing cytokines, consistent with previous studies demonstrating an inhibitory effect of the AhR on Th17 differentiation^26,27^ and suppression of Th2 differentiation by IL-21^28^. Conversely, the AhR inhibitor increased *IL21* expression compared to Th17 cultures. Although IL-21 can enhance the binding of AhR to the *IL22* promoter^29^, we observed an inverse correlation between *IL21* expression (Fig. 4A) and IL-22 production (Fig. 1B).

### Genes suppressed by the AhR

In addition to *IL21*, several other genes were identified that have not previously been reported to be AhR-regulated. In comparison to Th17-treated cultures, treatment with the AhR inhibitor CH223191 increased the expression of *ABI2* (1.6-fold), *CARD14* (1.5-fold), *CD160* (1.7-fold), *FOXH1* (1.5-fold), *LIG3* (1.5-fold), *MPHOSPH8* (1.4-fold), *PPP1R18* (1.6-fold) and *TMEM50B* (1.5-fold) (Supplementary Fig. S4). *ABI2* contributes to T cell receptor induced proliferation by enhancing signaling through the T cell receptor and providing costimulation^30^. On the other hand, CD160 inhibits human CD4 T cell proliferation and IFN-γ production by interacting with herpesvirus entry mediator^31^. FOXH1 is involved in TGF-β-dependent transcription by interacting with SMAD2 and SMAD4^32^. Thus, the AhR may suppress TGF-β function through inhibition of *FOXH1* expression. This may contribute to reduced levels of *RORC* observed in cultures treated with FICZ (Fig. 1). *CARD14* can activate NF-κB to induce pro-inflammatory cytokines, and gain of function mutations in *CARD14* are associated with psoriasis^33^. Circulating Th17 cells are increased in patients with psoriasis, and NF-κB blockade inhibits cytokine production from these Th17 cells^34^. It is intriguing to hypothesize that CARD14 suppression by AhR agonists can inhibit Th17 cell cytokine production in patients with psoriasis. Di Meglio, *et al*. has reported anti-inflammatory effects of AhR agonists in lesional skin of psoriasis patients^35^. The same study found that AhR-deficient mice have exacerbated skin inflammation in response to imiquimod, demonstrating that the AhR protects against experimental psoriasis.

Genes that were suppressed by FICZ treatment included *AIM1* (1.8-fold), *ANAPC1P1 (2*.*1-fold)*, *BASP1* (1.2-fold), *CCR3* (3.6-fold), *CD48* (2.0-fold), *PRR34* (1.6-fold), *PTMS* (1.8-fold), *SBF2* (1.6-fold) and *VTI1B* (1.6-fold) (Fig. 4). *BASP1* was previously shown to be expressed in human Th17 cells^36^, although its function in T cells is not known. CD48 is a costimulatory molecule that was found to be expressed in encephalogenitic T cells producing IL-17, IFN-γ and GM-CSF^37^. These data provide further support that AhR activation inhibits phenotypic changes in human CD4 T cells associated with Th17 cell differentiation. Overall, it remains to be determined if AhR-dependent genes are directly or indirectly regulated through AhR-mediated suppression of *RORC*.

### Genes upregulated by the AhR

Among the genes suppressed by CH223191 treatment were *BCL9L* (1.5-fold), *CD96* (2.1-fold), *GPR15* (3.9-fold), *GPR68* (2.2-fold), *GSG2* (1.9-fold) and *ITGAE* (1.3-fold) (Supplementary Fig. S4). CD96 is a member of the immunoglobulin superfamily with an ITIM-like domain, suggesting it may have an inhibitory effect on T cell costimulation^38^. *ITGAE* encodes for CD103, an E-cadherin receptor expressed on tissue-resident memory T cells^39^. *ITGAE* was found to be expressed by IL-17^+^ Foxp3^+^ T cells, indicating it can be used as a marker to identify Th17 cells that are transdifferentiating into Tregs^40^. On the other hand, CD103^+^ CD4 T cells express higher levels of IL-17, IFN-γ and TNF than CD103^-^ CD4 T cells in patients with ulcerative colitis^41^, suggesting this subset can drive intestinal inflammation. The upregulation of CD103 in response to TGF-β appears to play a role in the retention of memory T cells in mucosal tissues through its interactions with epithelial E-cadherin^39^. *In vivo*, AhR activation with TCDD was found to increase the number of splenic CD103^+^ CD11c^+^ dendritic cells^42^, suggesting the AhR can promote Treg differentiation through its effects on dendritic cells.

**Figure 5:**
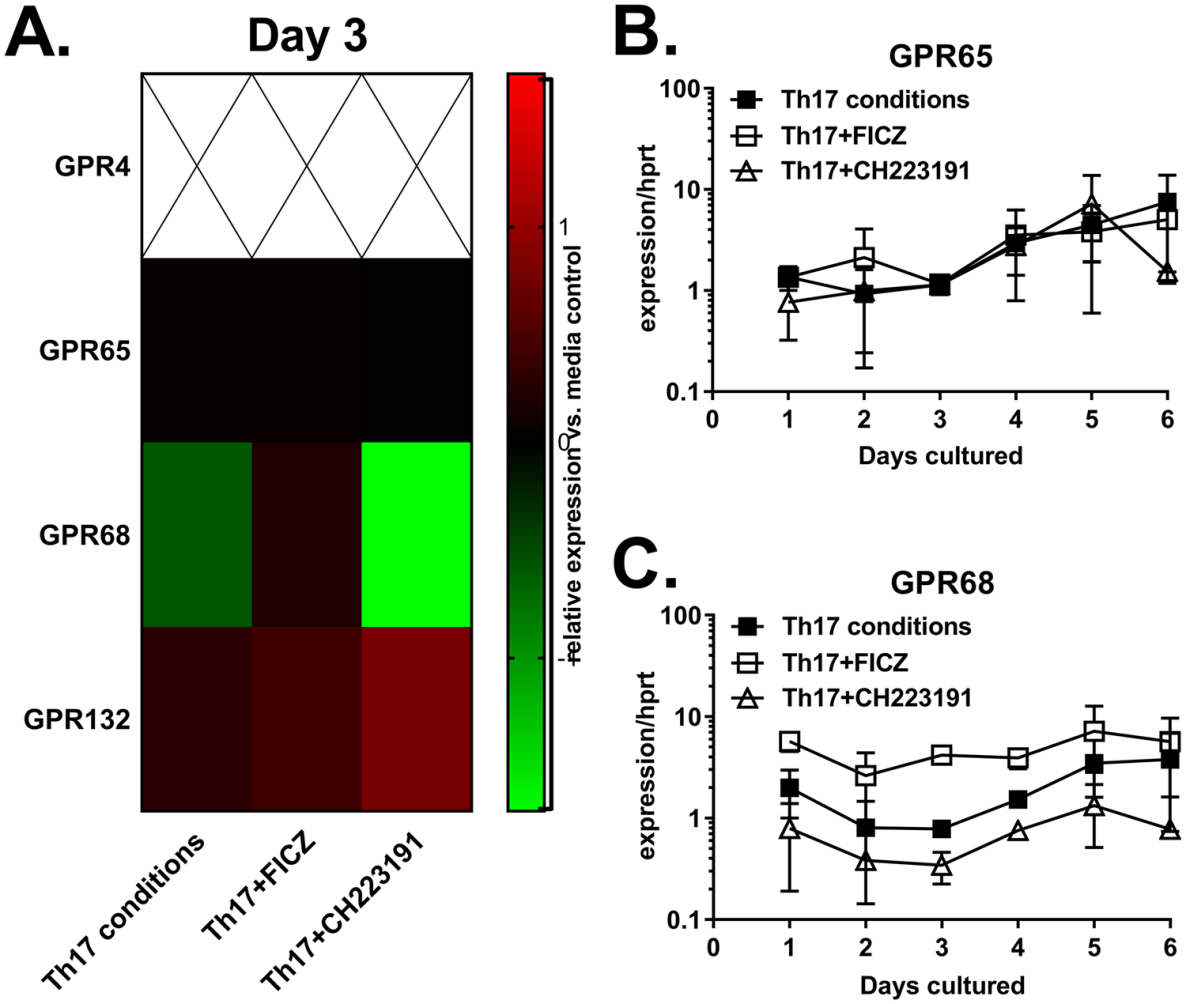
Expression of proton-sensing GPRs in Th17 cell cultures. (**A**) Heat map of proton-sensing GPRs from day 3 cultures. Data are from the same experiments described in Figs 3 and 4. Empty sections indicate that GPR4 was not expressed. (**B**) and (**C**) Expression of GPR65 and GPR68 in cultures containing Th17-inducing cytokines (Th17), measured by real-time PCR each day for 6 days, as indicated. Data are from the same experiments described in Fig. 1A.

Genes that were upregulated by FICZ treatment included *ANKRD13C* (4.4-fold), *CYP1B1* (3.3-fold), *PRCD* (4.6-fold), *S1PR4* (2.1-fold), *TREH* (3.7-fold) and *ZBTB21* (4.3-fold) (Fig. 4B). Of note, ANKRD13C is a molecular chaperone for G protein-coupled receptors (GPRs)^43^, several of which have been shown to regulate T cell function. Therefore, we hypothesized that the AhR regulates T cell cytokine production through GPR upregulation. In support, GPR15 was increased by FICZ and decreased by CH223191 in Th17 cultures (Supplementary Fig. S4). GPR15 is a colon-specific trafficking molecule enriched on peripheral Tregs^44^. Murine models have demonstrated a role for GPR15 in T cell-dependent colitis^45^, although most data suggests that GPR15 regulates gut homing rather than cytokine production from T cells. FICZ prevented the downregulation of *GPR55* and *GPR68* observed during Th17 differentiation (Figs 3, 5). GPR55 is a cannabinoid receptor expressed in immune cells, the GI tract and brain^46^. Activation of GPR55 increases intracellular calcium through G_q/11_ and phospholipase C^47^. Experimental colitis models have demonstrated that GPR55 agonists and antagonists are capable of protecting against intestinal inflammation^48,49^, demonstrating complex roles for GPR55 and cannabinoids in the gut. Currently, the impact of endogenous GPR55 signaling on T cell differentiation is not known, although it was shown to activate the NFAT transcription factor in transfected cells^50^, suggesting that GPR55 may influence T cell receptor signaling. GPR68, or ovarian cancer G-protein-coupled receptor 1 (OGR1), is a proton-sensing receptor activated by extracellular acidification^15^. This receptor is widely expressed in the immune system, small intestine, lungs and brain, among other tissues^51^. GPR68 activation increases intracellular Ca^2+^ concentrations through G_q/11_^52^. Since several chronic inflammatory diseases are associated with extracellular acidification^14^, the impact of GPR68 activation on Th17 cell cytokine production was further examined.

### GPR68 negatively regulates IL-22 and IL-10 production from differentiating CD4 cells

**Figure 6:**
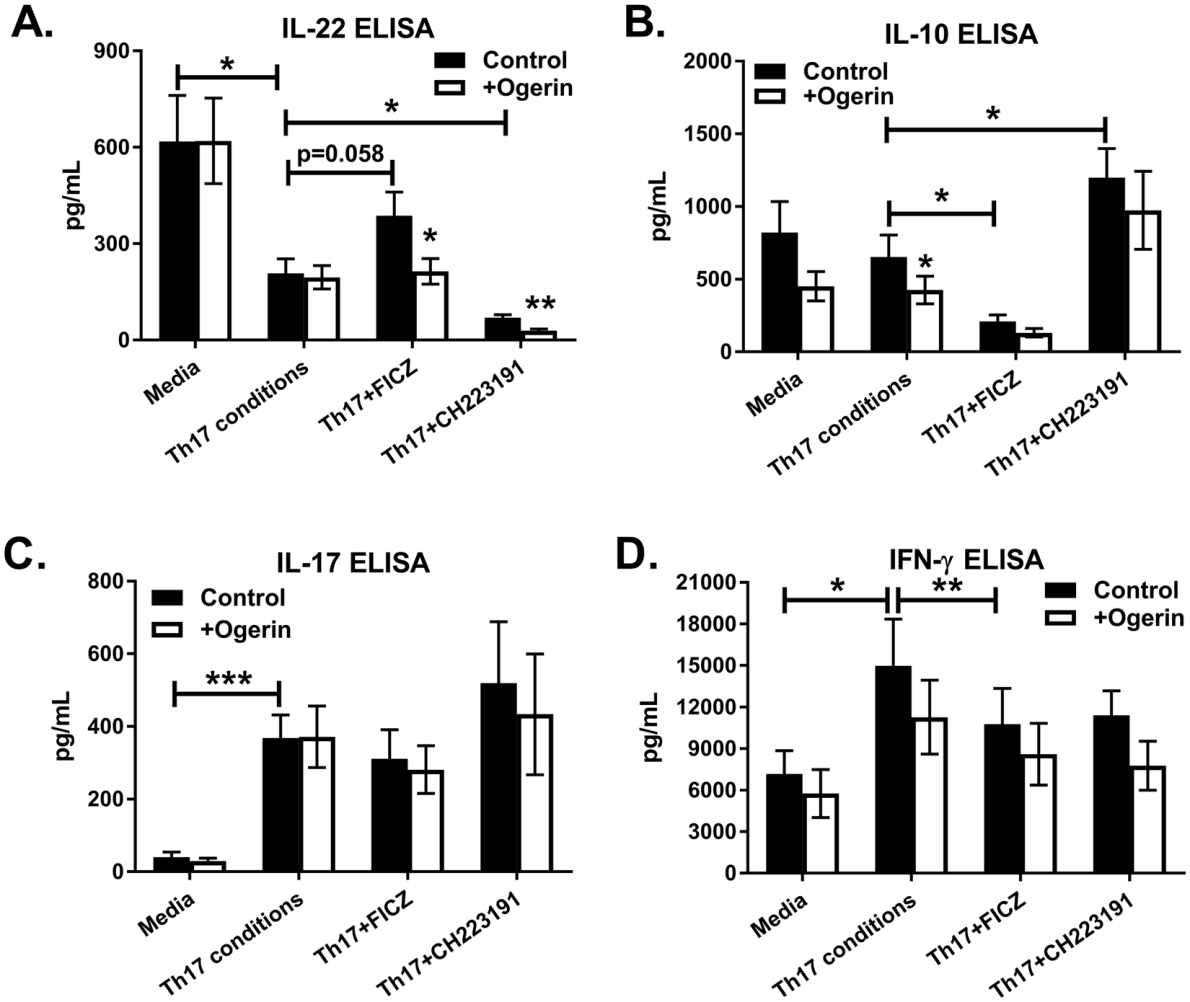
Enforced GPR68 stimulation inhibits IL-22 and IL-10 production from human CD4 T cells. Naïve CD4 T cells were cultured with Th17-inducing cytokines (Th17) for six days in the presence or absence of FICZ or CH223191, as indicated. Experimental groups were treated with (open bars) or without (closed bars) ogerin. On day 6,  IL-22 (**A**), IL-10 (**B**), IL-17 (**C**) and IFN-γ (**D**) were measured in culture supernatants. Shown are the Mean+/− SEM from four experiments with n = 8.

Among the four proton-sensing GPRs, only GPR68 was significantly increased by FICZ treatment (Fig. 5). Gaublomme *et al*. found that GPR65 is upregulated on pathogenic Th17 cells^53^; however, AhR modulators did not influence *GPR65* expression during the six day culture (Fig. 5). To determine the role of GPR68 activation in human Th17 cell differentiation, a lorazepam-derived positive allosteric modulator named ogerin was used^54^. Naïve CD4 T cells were cultured under Th17 conditions with or without AhR modulators, as described above. Treatment with ogerin suppressed IL-22 and IL-10 production in cultures treated with Th17-inducing cytokines, but had no effect on IL-17 (Fig. 6). To our knowledge this is the first demonstration of feedback inhibition on IL-22 production following AhR activation. This finding may have implications for inflammatory bowel diseases (IBDs), as IL-22 protects against chemical-induced colitis^10^. In addition, patients with IBD have increased *GPR68* expression in intestinal mucosa^55^. This study also found that endogenous *Gpr68* expression contributes to spontaneous intestinal inflammation in the *Il10*^−/−^ mouse model. Mice deficient in *Gpr68* are resistant to experimental autoimmune encephalomyelitis, correlating with fewer Th1 and Th17 cells^56^. These data demonstrate that a positive allosteric modulator of GPR68 suppresses IL-22 and IL-10 secretion from CD4 T cells in the presence of Th17-inducing cytokines.

### Gq-coupled receptors suppress spontaneous IL-22 secretion from human CD4 T cells

**Figure 7:**
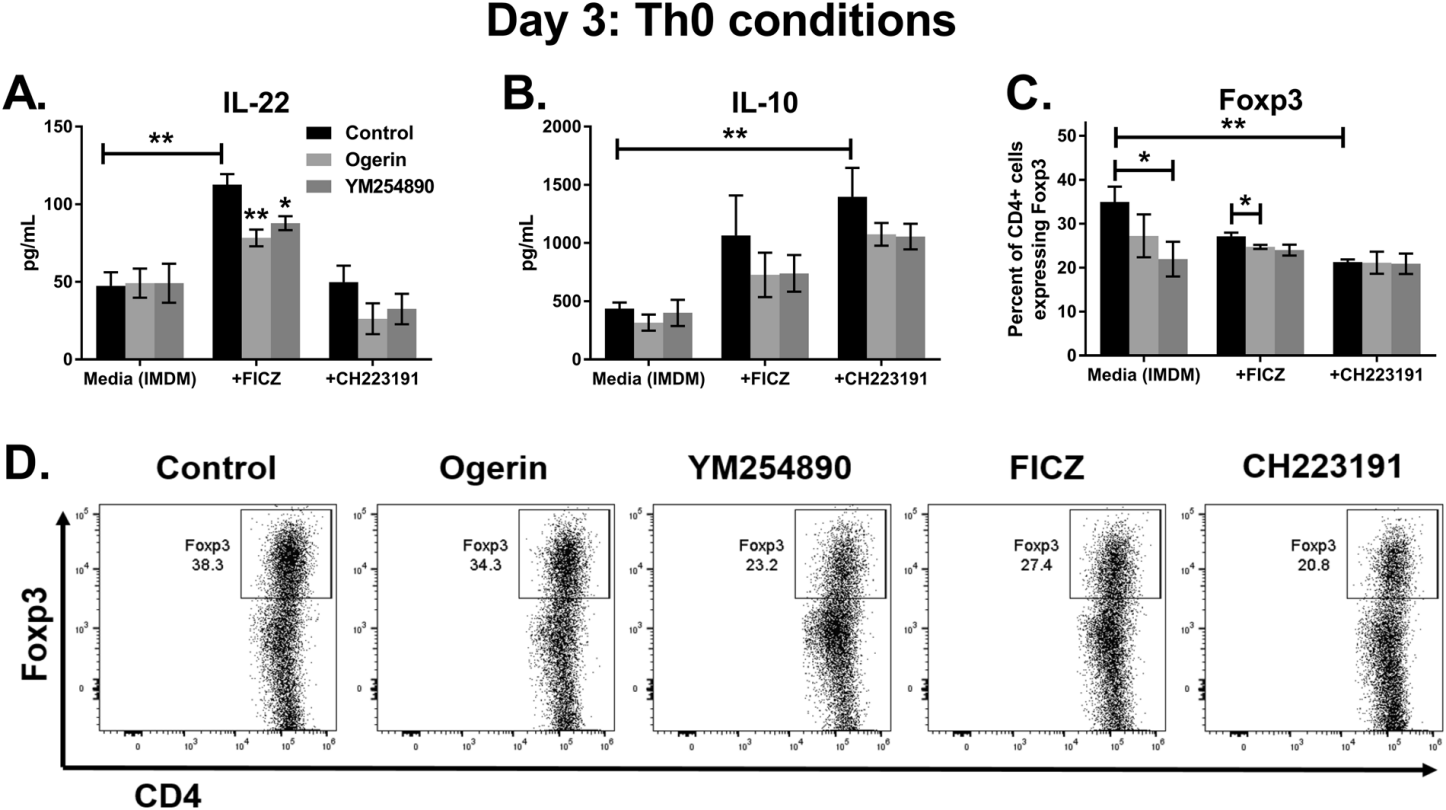
Gq-coupled receptors decrease IL-22 secretion and Foxp3 expression in human CD4 T cells. Naïve human CD4 T cells were cultured in IMDM, 10% FBS and anti-CD3/CD28 antibodies for three days. Experimental groups received FICZ, CH223191, ogerin or the Gα (q/11) inhibitor YM254890, as indicated. IL-22 (**A**) and IL-10 (**B**) were measured in culture supernatants by ELISA. (**C,D**). Day 3 flow cytometry staining for Foxp3, gated on CD4 T cells. Data are combined from two experiments with n = 5–6.

To determine if GPR68 regulates T cell function under non-differentiating (Th0) conditions, naïve CD4 T cells were cultured without exogenous cytokines in the presence of plate-bound anti-CD3 and soluble anti-CD28 antibodies for three days. FICZ treatment significantly increased IL-22 secretion compared to control medium (IMDM with 10% FBS; Fig. 7A). Ogerin partially suppressed the induction of IL-22 by FICZ (Fig. 7A), similar to cultures with Th17-inducing cytokines (Fig. 6A). The G_q/11_ receptor inhibitor YM254890 had a similar effect as ogerin, partially suppressing IL-22 induction by FICZ under Th0 conditions (Fig. 7A). This supports our hypothesis that AhR-dependent IL-22 production is regulated by Gq-coupled receptors. Inhibiting the AhR with CH223191 significantly increased IL-10 secretion compared to media alone (Fig. 7B). Although ogerin or YM254890 treatment resulted in a trend towards decreased IL-10, this effect was not statistically significant. We observed that 35 percent of CD4 T cells in the Th0 cultures expressed the regulatory T cell (Treg) transcription factor Foxp3 (Fig. 7C). Treatment with CH223191 or YM254890 significantly decreased the percent of CD4 T cells expressing Foxp3, suggesting a potential role for the AhR and Gq-coupled receptors in Treg differentiation. Overall, our data indicate the mechanism through which the AhR regulates human CD4 T cell function may be partially dependent on G_q/11_-coupled receptors such as GPR68 and GPR55. In future studies, it will be interesting to measure these receptors on *ex vivo*-isolated Th17 and Th22 cells.

## Conclusions

The regulation of cytokine production by human Th17 cells has clinical relevance for several chronic inflammatory diseases in which Th17 cells play a role. Interleukin-1β produced by innate immune cells supports pro-inflammatory functions of Th17 cells, in part by suppressing IL-10^5,57^. Our data demonstrate that AhR ligation with FICZ also suppresses IL-10 (Figs 1B and 6B), suggesting this may contribute to its pro-inflammatory effects. Murine models have demonstrated that pathogenic Th17 cells produce GM-CSF^58,59^. In human CD4 T cells, IL-23 suppresses GM-CSF production while IL-2 and IL-12 increase GM-CSF^60^. Consistent with this, we observed that *CSF2* significantly decreased when T cells were cultured with the combination of IL-1β, IL-6, IL-23 and TGF-β. In our model, AhR modulators had no effect *CSF2* expression. The role of T cell-derived GM-CSF in human disease remains to be clarified.

Cytokine production by CD4 T cells is accompanied by their upregulation of tissue homing receptors. Duhen *et al*. identified a subset of skin-homing memory T cells producing IL-22 but not IL-17^61^. This population of Th22 cells expresses CCR4, CCR6 and CCR10 in response to IL-6 and TNF stimulation. On the other hand, IL-17 was found to be produced by CCR4^+^ CCR6^+^ CCR10^-^ cells, demonstrating that expression of CCR10 can distinguish Th17 and Th22 subsets. These chemokine receptors were not regulated by FICZ or CH223191 in our study, suggesting a limited role for the AhR in tissue trafficking. Alternatively, the day 3 time point may not have been optimal to measure chemokine receptors. Our pathway analysis revealed several AhR-dependent genes to be involved in cell growth, development and proliferation. In the future, it will be interesting to examine the role of GPR68 in cytokine production by memory CCR4^+^ CCR6^+^ CCR10^+^ and CCR4^+^ CCR6^+^ CCR10^-^ CD4 T cell subsets.

In summary, we identified several gene targets of the AhR regulating CD4 T cell proliferation, differentiation and function. We further demonstrated that Gq-coupled receptors such as GPR55 or GPR68 may be involved in T cell cytokine production in response to AhR ligands. Our data showing a role for the proton-sensing receptor GPR68 in suppressing IL-22 and IL-10 production suggests that extracellular acidification may regulate T cell function through a receptor-dependent mechanism. Several chronic inflammatory diseases are associated with extracellular acidification including asthma, arthritis, atherosclerosis and tumors^14^. Gq-coupled receptors have been implicated in lupus and rheumatoid arthritis^62^. Further studies are warranted to investigate cell type-specific functions of GPR55 or GPR68 in chronic inflammatory diseases.

## Methods

### Th17 cell culture

Blood samples from healthy human volunteers were obtained from Zen-Bio, Inc., Research Triangle Park, NC. Each volunteer signed IRB or FDA informed consent forms validated by Pearl Pathways, LLC. Samples were processed according to Standard Operating Procedure managed Good Laboratory Practice protocols in compliance with all legal and ethical regulations. Peripheral blood mononuclear cells (PBMCs) were isolated from human buffy coat by density gradient centrifugation. Briefly, buffy coat was diluted 1:3 in PBS, overlayed onto Ficoll-Paque PLUS (GE Healthcare) and centrifuged at 400 × g for 20 min with the brake off. The cloudy interface containing PBMCs was washed with PBS, centrifuged (450 × g, 5 min) and resuspended in PBS with 2% FBS and 1 mM EDTA. Naïve CD4 T cells were purified using a commercially available kit (StemCell Technologies, Vancouver, BC, Canada). Following purification, cells were resuspended in RPMI with serum replacement factor 3 (Sigma-Aldrich, St. Louis, MO), anti-CD28 clone CD28.2 (2 ug/mL; Biolegend, San Diego, CA), L-glutamine and penicillin-streptomycin. Cells were enumerated using trypan blue exclusion, and 0.5–1.0 × 10^6^ were seeded in 24 well plates pre-coated the day before with anti-human CD3 clone OKT3 or UCHT-1 (10 ug/mL; Biolegend). In some experiments, 0.1 × 10^6^ cells were seeded in 96 well plates pre-coated with anti-CD3 clone UCHT-1 (10 ug/mL). CD4 T cell purity ranging from 90–98 percent was verified by flow cytometry.

Recombinant human cytokines were purchased from R&D Systems. Th17 conditions included culturing naïve CD4 T cells with IL-6 (30 ng/mL), TGF-β (2 ng/mL), IL-1β (10 ng/mL) and IL-23 (10 ng/mL). Th22 culture conditions were IL-1β, IL-6, IL-23, Galunisertib (10 uM), anti-IL-4 (0.5 ug/mL), anti-IFN-γ (0.5ug/mL) in IMDM with 10% fetal bovine serum. Th0 culture conditions, or Media, were plated-bound anti-CD3 and soluble anti-CD28. Additional treatments included FICZ (200 nM; Sigma-Aldrich), CH223191 (4 uM; Sigma-Aldrich), ogerin (10 uM; Tocris Bioscience) and YM254890 (1 uM; Adipogen Life Sciences).

### RNA quantitation and RT-PCR

Total RNA was purified from T cell cultures using TRIzol reagent (Ambion) and quantitated by spectrophotometry (NanoDrop Lite, Thermo Scientific). For quantitative real-time PCR, 1ug of RNA was used as a template for reverse transcription into cDNA (iScript, Bio-Rad). PCR reactions were performed using TaqMan Gene Expression Master Mix and commercially available primers labeled with FAM-MGB (Applied Biosystems). PCR reactions were run on a QuantStudio 3 (Applied Biosystems) with the following conditions: 50 C for 2 min, 95 C for 10 min, and 40 cycles of 95 C for 15 sec and 60 C for 1 min. Target gene expression was normalized to the housekeeping gene *HPRT* and analyzed using the delta-delta Ct method^63^.

### RNA-Seq and data analysis

Naïve CD4 T cells from peripheral blood of six healthy human volunteers were cultured in serum-free RPMI and serum replacement factor 3 (Sigma-Aldrich) under four experimental conditions: (1) anti-CD3 and anti-CD28 antibodies (control medium), (2) Th17 differentiation conditions (IL-6, TGF-β, IL-1β, IL-23), (3) Th17 + FICZ (AhR agonist) and (4) Th17 + CH223191 (AhR antagonist). On day 3, total RNA was extracted from each of the 24 samples using TRIzol, and all samples had RNA integrity numbers greater than 8. Libraries were prepared from 1 μg of total RNA per sample using the TruSeq stranded mRNA kit (Illumina, Inc., San Diego, CA), according to the manufacturer’s instructions. Library quality was assessed by electrophoretic analysis on the Agilent Bioanalyzer 2100 system. RNA-Seq libraries were sequenced in a 2 × 50 paired-end design on an Illumina HiSeq1500.

The number of reads passing filter per library ranged from 12–24 million read pairs (24–48 million total reads. Raw reads were imported from the Illumina HiSeq1500 using bcl2fastq2 v2.17.1.14 (Illumina). Reads were trimmed to remove adapter content and low quality base calls, with reads trimmed to a length of less than 25 bases removed from further analysis using Trimmomatic v0.36^64^. Quality of reads was assessed before and after trimming using FastQC v0.11.5 (Babraham Institute, Cambridge, England). Reads were aligned to the human reference genome GRCh38 using HISAT2 v2.0.4^65^, and the resulting bam files were sorted and indexed using SAMtools v 1.3.1^18^. Differential gene expression was established using DESeq. 2^66^ with a False Discovery Rate (FDR) threshold of 0.1. Three separate comparisons were made: Th17 versus control; Th17 + FICZ versus Th17; and Th17 + CH223191 versus Th17. In each case, expression ratios were calculated as the expression of genes in the first-named sample group divided by the expression in the second-named sample group. A script showing the calls to all tools, with all parameter settings, is uploaded (pipeline.sh). Expression ratios shown for each treatment group in heat maps is normalized to the media control (Figs 2–4 and 6).

### Flow cytometry

Following T cell culture, cells were resuspended in media containing phorbol 12-myristate 13-acetate (PMA, 50 ng/mL, Fisher Scientific), ionomycin (750 ng/mL, Fisher Scientific) and brefeldin A (5 ug/mL, Tocris Bioscience) for 4 hours. Cells were then fixed, permeabilized and stained with fluorescent-labelled antibodies for CD4, FoxP3, IL-17 and IL-22 using FoxP3 Fix/Perm Buffer Set (Biolegend). Cells were analyzed on a Novocyte 3000 flow cytometer (Acea Biosciences, Inc.), with data analyzed using FlowJo software.

### Data availability

Raw data are deposited at the National Center for Biotechnology Information’s Gene Expression Omnibus (NCBI GEO) with accession number GSE102045.

### Statistical analysis

Data is plotted as the mean +/− standard error of the mean (SEM). Two-tailed Student’s t-tests were used to compare experimental and control groups, with asterisks representing statistically significant differences (*p < 0.05, **p < 0.01, ***p < 0.001). Pairwise analysis was performed where appropriate.

## Electronic supplementary material

Supplementary information
