# Supplementary material for: Cytokine Regulation in Human CD4 T Cells by the Aryl Hydrocarbon Receptor and Gq-Coupled Receptors
Source: Sci Rep. 2018 Jul 19;8:10954. doi: 10.1038/s41598-018-29262-4 (PMC6053392; doi:10.1038/s41598-018-29262-4)
Supplement: Supplementary file 1 — Supplementary information [file 41598_2018_29262_MOESM1_ESM.docx]

**Cytokine Regulation in Human CD4 T Cells by the Aryl Hydrocarbon Receptor and Gq-Coupled Receptors**

Jeremy P. McAleer^1*^, Jun Fan^2^, Bryanna Roar^1^, Donald A. Primerano^2^ and James Denvir^2^

^1^Department of Pharmaceutical Science and Research, Marshall University School of Pharmacy, Huntington, WV 25755

^2^Department of Biomedical Sciences, Joan C. Edwards School of Medicine, Marshall University, Huntington, WV 25755

*Corresponding author: Jeremy P. McAleer, Ph.D.

Phone: 304-696-7336

Fax: 304-696-7309

e-mail: mcaleer@marshall.edu

**Supplementary Information**

**
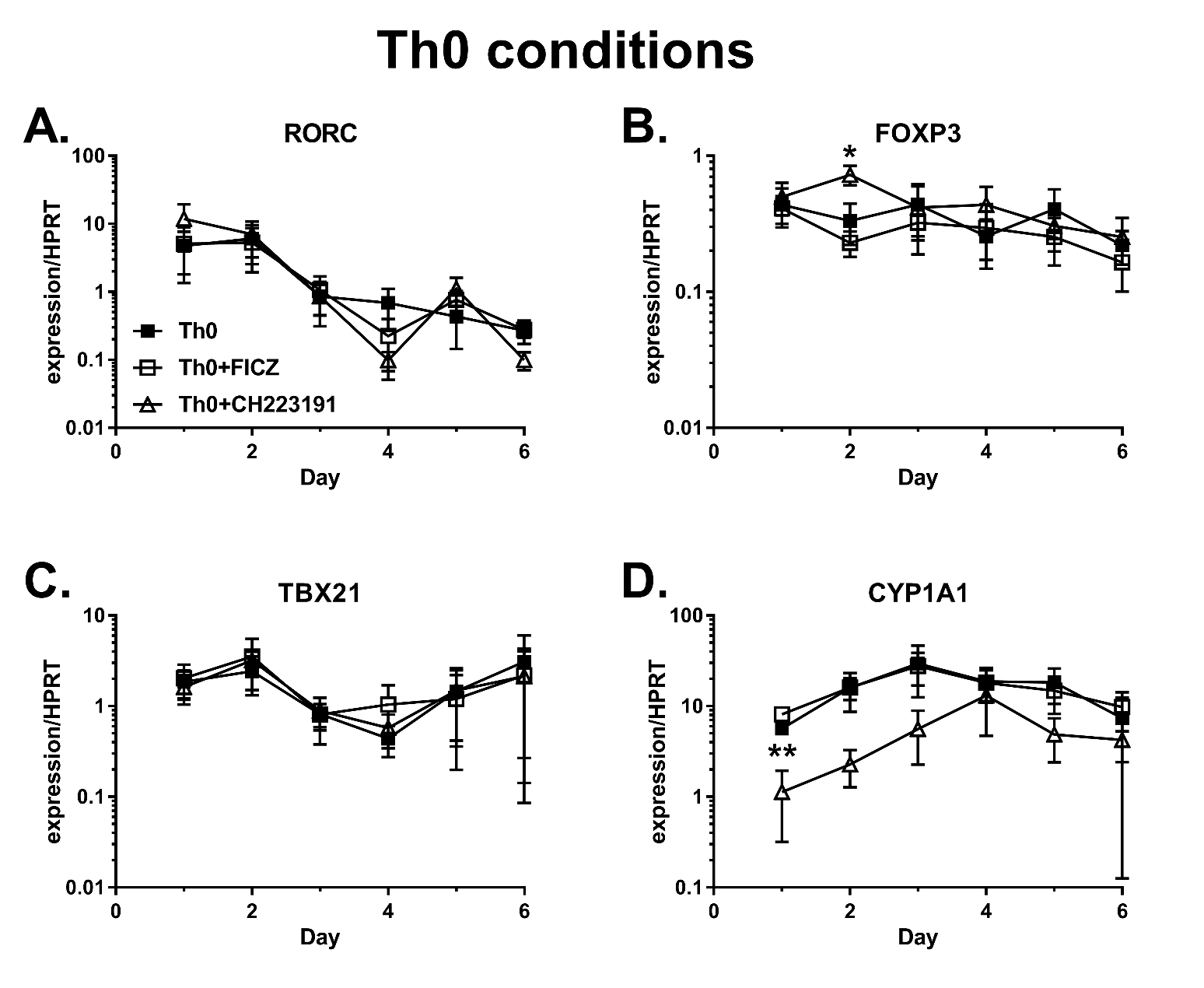
**

**Supplementary Figure S1. Effect of AhR modulators on human CD4 T cell differentiation.** Naïve human CD4 T cells purified from peripheral blood of healthy volunteers were cultured in RPMI with serum replacement factor under Th0 conditions (plate-bound anti-CD3, soluble anti-CD28) for six days in the presence of FICZ (200nM) or CH223191 (4uM), as indicated. RNA was isolated each day and analyzed for gene expression of *RORC*, *FOXP3*, *TBX21* and *CYP1A1* by real-time PCR, normalized to the housekeeping gene *HPRT*. Data are combined from 3 independent experiments using different donors and shown as mean +/- SEM.


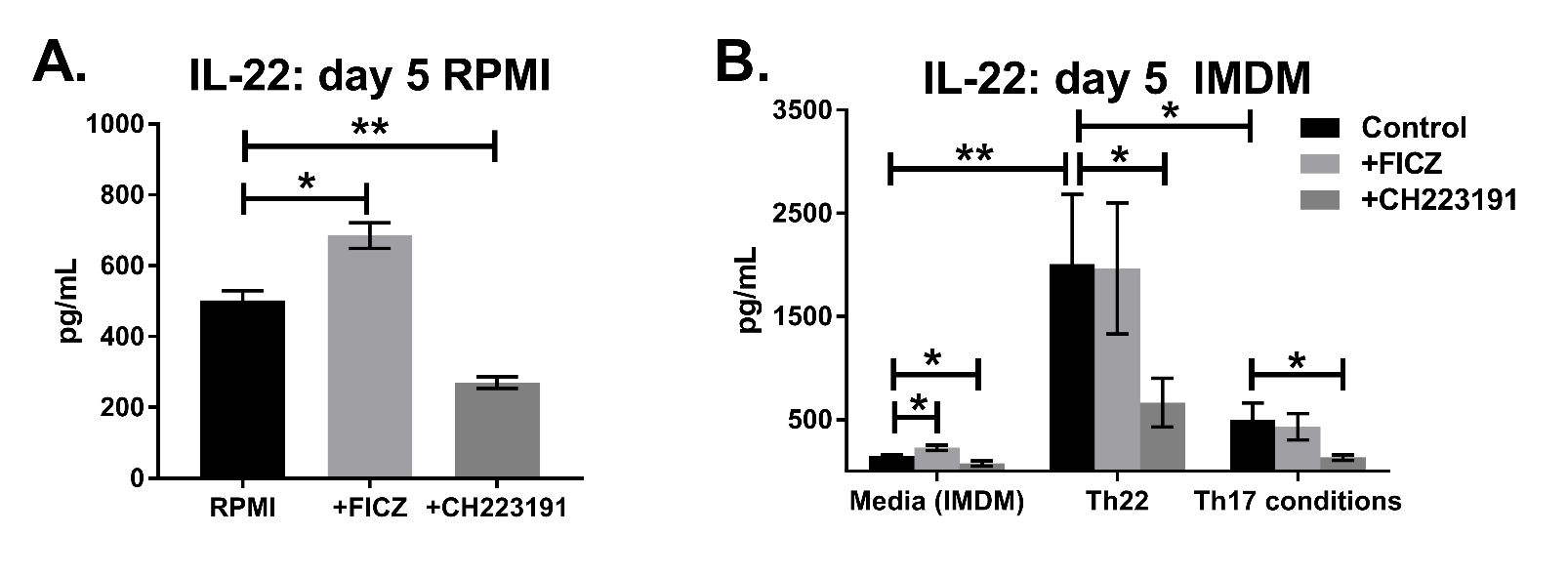


**Supplementary Figure S2. Effect of AhR modulators on IL-22 production by CD4 T cells.** Naïve CD4 T cells were cultured in serum-free RPMI (A) or IMDM with 10 percent FBS (B) for five days. Experimental groups were treated with FICZ, CH223191, Th22 conditions (IL-1β, IL-6, IL-23, Galunisertib 10uM) or Th17 conditions (IL-1β, IL-6, IL-23, TGF-β), as indicated. Shown are IL-22 levels in culture supernatants on day 5, measured by ELISA. Treatments were performed in triplicate from one (A) or two (B) independent experiments. Paired t-tests were used for statistical analysis.


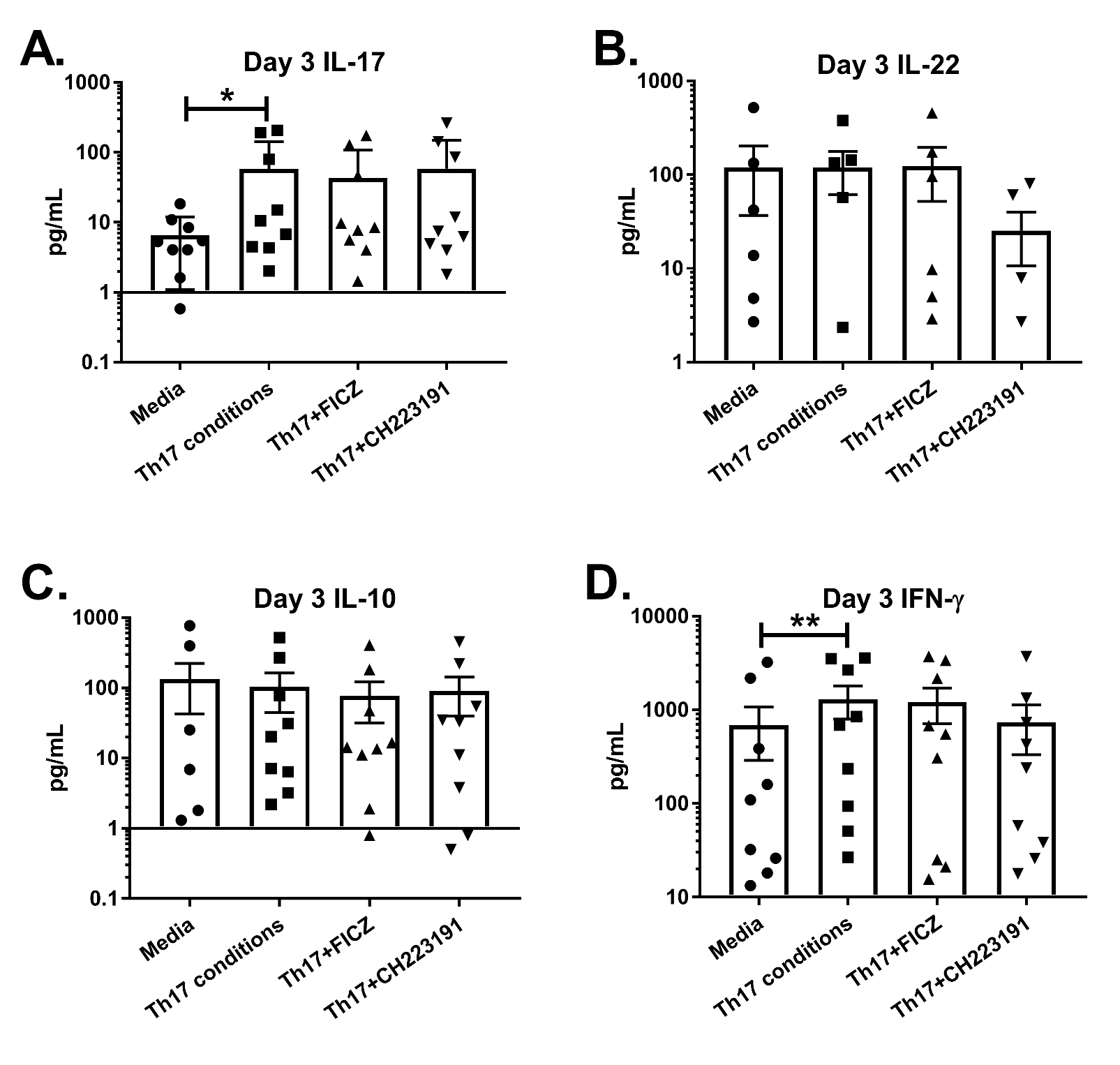


**Supplementary Figure S3. CD4 T cell cytokine production in the presence of AhR modulators for three days.** Naïve (CD45RO^-^) human CD4 T cells were cultured with Th17-inducing cytokines in the presence or absence of FICZ or CH223191, as indicated. Cytokines were measured in culture supernatants on day 3. Data are from the same donors used in Figure 1B. Asterisks represent statistically different values between the indicated groups, determined by pairwise analysis.


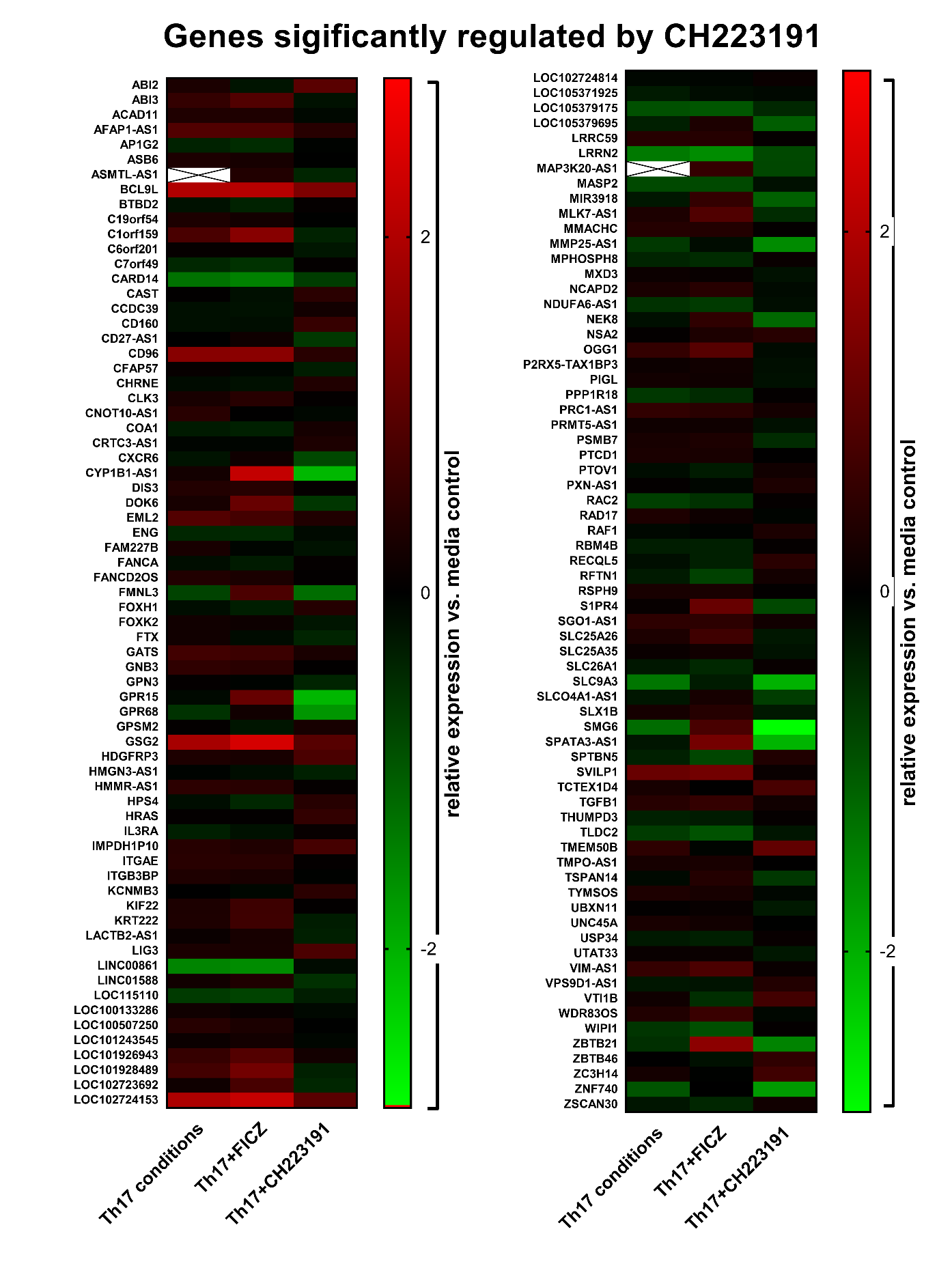


**Supplementary Figure S4. Heat map of genes expressed at significantly different levels between Th17 and Th17+CH223191 groups (day 3).** Relative gene expression is normalized to the media control (0). Empty sections indicate no detectable gene expression for the corresponding treatment.
